# Use of smartphone and perception towards the usefulness and practicality of its medical applications among healthcare workers in Saudi Arabia

**DOI:** 10.1186/s12913-019-4523-1

**Published:** 2019-11-12

**Authors:** Mostafa A. Abolfotouh, Ala’a BaniMustafa, Mahmoud Salam, Mohammed Al-Assiri, Bader Aldebasi, Ibraheem Bushnak

**Affiliations:** 1 Research Training & Development Section, King Abdullah International Medical Research Center (KAIMRC), King Saud bin-Abdulaziz University for Health Sciences (KSAU-HS), King Abdulaziz Medical City, Ministry of National Guard- Health Affairs, Riyadh, 11426 Saudi Arabia; 2 Research Office, King Abdullah International Medical Research Center (KAIMRC), King Saud bin-Abdulaziz University for Health Sciences (KSAU-HS), King Abdulaziz Medical City, Ministry of National Guard- Health Affairs, Riyadh, 11426 Saudi Arabia

**Keywords:** Attitude, Healthcare facility, Health-related applications, Pattern of use, Smartphone use

## Abstract

**Background:**

In Saudi Arabia, healthcare industry is undergoing major expansions to meet the demand of rapidly growing healthcare needs. The aims of this study were; (1) to assess the pattern of smartphone use in healthcare facilities, and (2) to determine perception towards its use among healthcare workers.

**Method:**

A cross-sectional survey of 351 healthcare workers (HCWs) at King Abdulaziz Medical City (KAMC) in Riyadh, Saudi Arabia was conducted, from October to November 2016, using a previously validated perception domain to measure perception towards usefulness (5 statements) and practicality (5 statements) of smartphones in clinical settings. Pattern of use of smartphones and health-related applications in healthcare facilities was also investigated. Logestic regression models were applied to identify the predictors of smartphone use and installation of health-related applications for use in healthcare facilities. Significance was considered at *p*-value of < 0.05.

**Results:**

Utilization rate of smartphone was 42.3%, and only 6.1% of all healthcare providers reported always using applications in their practice. Reasons for use were: as a source of drug information (69.8%), for disease diagnosis (56.4%), to access medical websites (42.5%), to review guidelines and protocols related to healthcare (34.1%), for procedure documentation (23.5%), and as a source of patients education materials (22.3%). Perceptions of HCWs towards smartphone use was less than satisfactory (Overall percentage mean score = 60.4 ± 18.7), with only 11.6% reporting positive perception. After adjusting for possible confounders, the total perception mean score was a significant predictor of both smartphone use (β = 0.033, *p* < 0.001) and medical applications installation (β = 0.033, p < 0.001). Installation of medical applications was also predicted by being a physician (β = 0.008, *p* = 0.024).

**Conclusion:**

Smartphone utilization in healthcare facilities by HCWs in Saudi Arabia is low. This could be attributed to their less than satisfactory level of perception towards its use. Smartphone use and installation of medical applications for use in health facilities were predicted by perceived usefulness and practicality of its use. Intervention from higher health authorities is necessary to enforce the importance of smartphone use in clinical practice. Conduction of further studies on the impact of smart phone use on the healthcare quality in Saudi Arabia is recommended.

## Background

Information technology (IT) softwares in healthcare provide clinicians with health-related information and tools, such as clinical decision supportive systems. These softwares are supposed to reduce potential medical errors in any healthcare facility and improve the quality of patient care [1]. The rising demand to improve the healthcare processes via technical products and services has resulted in a huge development of wireless technologies worldwide [2].

Smartphone has started as a multi-functional mobile phone with advanced features. Some applications in smartphones are health-related, and may be useful in a wide variety of clinical aspects. Proper utilization of these applications vary in degrees of success, and this is based on being easy-to-learn and use [3]. A previous study reported that the utilization of smartphones had provided practitioners with immediate access to medical and health information, and this had a positive impact on a healthcare system. This technology has reduced numbers of medical errors and improved decision-making, thus improving the telemedicine communication between the hospital staff [4].

Smartphone applications have been widely accepted as training and information tools. The percentage of health professionals using smart phones has risen from 66 to 90% in 2012 [5]. A digital survey examining smartphone and its application use was conducted by Accreditation Council for Graduate Medical Education (ACGME) in California, showed that more than 85% of respondents used a smartphone, of which the iPhone was the most popular (56%). Over half of the respondents reported using applications in their clinical practice; the most frequently used application types were; drug guides (79%), medical calculators (18%), coding and billing applications (4%) and pregnancy wheels (4%). The most commonly requested application types were textbook/ reference materials (average response: 55%), classification/ treatment algorithms (46%) and general medical knowledge (43%) [6].

Davis in 1989 [7] formulated the Technology Acceptance Model (TAM) as a mechanism to explain and predict consumer’s acceptance of information technology and information system (IT/IS) [7]. There are originally five elements of TAM: perceived usefulness (PU), perceived ease of use (PEOU), attitude toward using, behavioral intention (BI) to use, and actual system use [2, 8]. The TAM model suggests that the decision to adopt a system is based on two main factors: Perceived Usefulness (PU) and Perceived Ease of Use (PEOU). PU is the extent a person feels that utilization of a specific technology would improve his/her job performance, while PEOU refers to the degree a person feels that utilization of a particular technology requires no effort [2, 7].

In Saudi Arabia, the healthcare industry is undergoing major expansions to meet the demand of rapidly growing healthcare needs. A user-acceptance study utlizing a TAM based questionnaire was recently conducted in Al-Qassim focusing on the perception and readiness of health providers towards mobile health applications [9]. However, locally conducted studies didn’t fully measure the combined factors affecting perception, attitude and practice using the TAM. On other side, another study was counducted in Almadinah Almunawwarah trying to assess patterns of smart phones usage among female medical students which focused on female population and assessed the side effects of smart phones [10]. At King Abdulaziz Medical City (KAMC), it is estimated that the number of healthcare providers exceeds 7000 between the central, eastern and western regions. However, rules and regulations regarding using smartphones at hospital by healthcare providers in Saudi Arabia have not yet existed. Accordingly, it was expected that the feedback provided by these professionals would provide better undertsnading of the efficiency and pacticality of a number of currently used health applications. Findings would contribute in assessing the needs for upcoming development of health related applications for staff education, thus providing instruction and guidence for optimal use of smart phone’s apps to enhance clinical practice. The aims of this study were; (1) to assess the pattern of smartphone use in healthcare facillities [time, frequency and purpose behind using health-related applications in smart phones], (2) to determine perception towards its use and (3) to identify factors influencing the usage of smartphone in clinical practice and installation of medical applications, among healthcare workers in a tertiary care setting.

## Methods

### Study area/setting

King abdulaziz medical city (KAMC), Ministry of National Guard-Health Affiars (MNG-HA), in Riyadh, is a distinguished healthcare provider, with the bed capacity of 1501 beds in addition to 25 beds allocated for expected surgical operations for admission of emergency cases. Since its inauguration in February 2001; and within a short period, KAMC has passed the requirements for accreditation under the (JCI) Joint Commission International standards with excellent performance in December 2006.

### Study subjects

All healthcare workers (HCWs) working in departments of medicine, nursing, laboratories, respiratory therapy, radiology, nutrition, paramedics, clinical pharmacists, auxiliiaries, sterilizing unit technicians, infection control practitioners.

### Study design

Cross sectional survey.

### Sample size and sampling technique

The Accreditation Council for Graduate Medical Education (ACGME) in California found that 56% of medical providers use smart phone applications in their clinical practice [6]. Assuming a 50% favourable perception, a margin of error of 5%, and 95% confidence level, the estimated sample size that would statistically be convenient in this study was 378 participants. To compensate for an estimated 25% drop out rate due to incomplete surveys and withdrawals, 470 surveys were distributed. Those who responded were 351 HCWs, with a response rate of 75%.The nature of the project design entitled a convenient sampling technique. This non-probabilty sampling approach voluntarily involved HCWs who were available to make the sample a better representative of the entire population of KAMC in Riyadh (~ 4000 HCWs). Careful attention was made to ensure various disciplines (medicine, nursing, etc.) participate in comparable sample sizes.

### Data collection

The survey was composed of three main sections:

1. Demographic characteristics of the study sample: A number of sample characteristics were collected as independent variables or exposures. These include; gender (Male / female), age (in years), nationality (Saudi/Non Saudi), education (BSN/ MSN/PHD /Tech /Diploma), occupation (Employee / Student), job title (Physician, Nurse, etc.), discipline (Medicine, Nursing etc.), and experience (in years),
2. Pattern of usage and purpose domain [4, 6, 11]: Possesion of smart phones, health-related applications or any other portable devices, frequency of usage, time spent in hours daily, etc..
3. Perceived Usefulness and Perceived Ease of Use. A previously validated domain that measures the perception towards the usefulness (5 statements) and practicality (5 statements) of smart phones in clinical settings was used [7, 9]. Perception score was calculated for the responses to the 10 statements by using 4-point Likert type scale ranging from 0 to 3 points; 3 points for strongly agree, on positive attitude sentence, to 0 point for “strongly disagree”. The total score for each HCW was calculated by summing scores for all responses, and then a percentage score was calculated. This percentage score was categorized into positive (> 75%), neutral (50–75%) and negative (< 50%) perception.

The investigators distributed an annonymous self administered English based survey inside an envelope with a cover letter. Each envelope was handed to the HCW at his/her department. HCWs are Saudis and expatriates of different nationalities, with Arab and non-Arab speakers, yet English language is the official language of communication among the HCWs at KAMC. Study particpants were expected to fill the survey and return it in the envelope sealed with no identifiers. The cover letter served as the front page that explained the purpose of the study and invited the HCW to particpate voluntarly and at his/her own leasure. The envelope was not recognized by the hospital or investigator. The cover letter assured the particpant that his/her feedback wont affect their work evaluation, work status, and salary. The collection of data was framed with confidentiality in a matter where the participant’s name and/or contact information were not identified or traced by anyone. No written consent was sought as approved by the IRB. The study was approved by the IRB of the Ministry of National Guard-Health Affairs (Ref. # RC16/002/R). This study was conducted in accordance with the Declaration of Helsinki. To test the feasibility of the study and the reliability and validity of data collection tool, a pilot study was performed on 20 randomly selected participants who were subsequently excluded from the main study.

### Data analysis

SPSS software Ver. 24 was used for data entery and analysis. Descriptive statistics such as; mean, standard deviation, frequency and percentages of all independent variables were used. Analytic statistics were uded to test associations of the HCWs’ perception towards smartphone use with both smartphone use and instalation of medical applications. Chi-square test was used for qualitative data, while student t-test and ANOVA were applied for quantitative data. To predict the significant predictors of HCWs’ smartphone use and installation of medical applications, logestic regression analyses were applied, with age group, nationality, education level, marital status, job title, working experience in years and total perception mean score as the independent variables. Significance was considered at *p*-value < 0.05.

## Results

A total of 351 healthcare workers responded to a survey to assess their perception towards the use of smartphone in clinical practice, with a response rate of 93%. The majority of those were non-Saudis (90.3%), married (55.2%) female (88.3%), nurses (76.6%), aged 30 to 40 years (40.5%), and working in medical departments (49.7%), Table 1.

**Table 1:** Demographic characteristics of healthcare workers

Characteristics	No (*n* = 351)	%
Gender		
Male	41	11.7
Female	310	88.3
Age group (yrs)		
20–29	86	25.6
30–39	136	40.5
40+	114	33.9
Mean (SD)	36.1 ± 8.7	
Education		
Diploma	89	25.7
BSN	247	71.4
MSN/PHD	10	2.9
Nationality		
Saudi	34	9.7
Non-Saudi	317	90.3
Marital status		
Single	145	41.7
Married	192	55.2
Divorced/ Widowed	11	3.1
Job title		
Physician	8	2.3
Nurse	268	76.6
Pharmacist	2	0.6
Lab. technician	29	8.3
Others	43	12.2
Workplace		
Medical	172	49.7
Surgical	21	6.1
Lab.	34	9.8
Pharmacy	1	0.3
Others	118	34.1
Work experience (in years)		
< 10	160	47.2
10-19	127	37.5
20 or more	52	15.3
Mean (SD)	11.6 ± 7.3	

### Ownership and use of smartphone

Almost all healthcare workers owned one or more smartphones (96.6%), but only 42.3% utilize it in healthcare practice. Less than two-thirds of all healthcare providers (60.1%) reported using other appliances such as; IPAD (43.6%), tablet (7.5%) and others (10.3%), and less than one-half installed applications to use in clinical practice (45.5%). Regarding the frequency of use of smartphone, only 6.1% of all healthcare providers reported always using the application in their practice, and 26.2% of them using it sometimes.The reasons for use of smartphone in healthcare practice were: as a source of drug information (69.8%), for disease diagnosis (56.4%), to access medical websites (42.5%), to review guidelines and protocols related to healthcare (34.1%), for procedure documentation (23.5%), and as a source of patients education materials (22.3%), Table 2.

**Table 2:** Ownership and utilization of smartphones by healthcare workers

	No.	%
**Own smartphone?**^a^		
None	12	3.4
Iphone	169	48.4
Android	168	48.2
Nokia	4	1.2
Blackberry	2	0.6
Others	5	1.4
**Utilization of smartphone in healthcare**	146	42.3
**Use of other appliences**^a^		
No	127	39.9
Tablet	24	7.5
IPAD	139	43.6
Others	33	10.3
**Installed medical applications?**	157	45.5
**Frequency of use**	**(*N*** **= 309)**	**%**
Never	142	46.0
Rarely	67	21.7
Sometimes	81	26.2
Always	19	6.1
**Reasons for use**^**a**^	**No (*n*** **= 179)**	**%**
Drug information	125	69.8
Clinical score systems (coma scale, APACHE, pain score …)	33	18.4
Disease diagnosis	101	56.4
Procedure documentation	42	23.5
Access medical web sites	76	42.5
Review guideline and protocol related to healthcare	61	34.1
Patients’ education materials	40	22.3
Others	12	6.7

^a^--data are not mutually exclusive

### Perception towards importance of smartphone use

Table 3 shows that beliefs of healthcare providers in smartphone use in terms of usefulness and practicality was less than satisfactory (Overall percentage mean score = 60.4 ± 18.7), only 11.6% reporting positive attitude. Percentage mean scores of 58.6 ± 19.7 and 62.5 ± 19.9 were shown for usefulness and practicality respectively, with only 10.2 and 12.3% reporting positive attitude towards the importance of smartphone use.

**Table 3:** Perception of healthcare workers towards usefulness and practicality of use of smart phone in clinical settings

	Perception towards smartphone use in healthcare	Strongly disagree	Disagree	Agree	Strongly agree
		No.(%)	No.(%)	No.(%)	No.(%)
	(A) *Usefulness*				
1	Medical apps on smart phone improve my tracking of patient condition and performance	20(6.2)	67(20.6)	212(65.2)	26(8.0)
2	Medical apps on smart phone save the time and efforts of healthcare provider	18(5.5)	60(18.4)	213(65.3)	35(10.8)
3	Medical apps on smart phone rapidly retrieve of the information from the patient.	19(5.9)	79(24.7)	197(61.6)	25(7.8)
4	Medical apps on smart phone allow healthcare provider to follow up the patient condition from outside of the hospital	25(7.7)	86(26.6)	181(56.0)	31(9.7)
5	Medical apps on smart phone enable healthcare provider to get the information of the patient quickly (through the software)	19(5.9)	51(15.8)	216(67.1)	36(11.2)
	Percentage mean score	58.6 ± 19.7			
	*(B) Practicality*				
6	Learning to operate and use medical apps on smart phone would be easy for me	15(4.6)	29(9.0)	234(72.2)	46(14.2)
7	I would find it easy get medical apps on smart phone to do what I want it to do	16(5.0)	55(17.1)	218(67.7)	33(10.2)
8	My interaction with medical apps on smart phone would be clear and understandable	16(5.0)	45(13.9)	232(71.8)	30(9.3)
9	I would find medical apps on smart phone to be flexible to interact with	16(4.9)	39(12.0)	236(72.8)	33(10.3)
10	It would be easy for me to become skillful at using medical apps on smart phone	19(5.8)	44(13.5)	224(68.9)	38(11.8)
	Percentage mean score	62.5 ± 19.9			
	Overall percentage mean score	60.4 ± 18.7			
		Negative	Neutral	Positive	
	*Levels of perception toward smartphone use*	No.(%)	No.(%)	No.(%)	
	Usefulness	82(26.0)	201(63.8)	32(10.2)	
	Practicality	54(16.9)	226(70.8)	39(12.3)	
	Overall perception of smartphone use	50(16.0)	226(72.4)	36(11.6)	

### Association between perception towards smartphone use and its use

Table 4 shows the association between the level of perception to smart phone use and prevalence of its use and installation. It shows that the prevalence of smartphone use was 26% among subjects with negative perception towards its use. This figure was doubled (43.2%, OR = 2.17, *p* = 0.025) and tripled (74.3%, OR = 8.22, *p* < 0.001), among those with neutral and positive perceptions respectively. This finding was evident for both perception domains; usefulness and practicality. Likewise, the prevalence of installation of medical applications was 28% among HCWs with negative perception towards smartphone use, and this figure was doubled and tripled as we shifed from those with negative perception (20.0%) to neutral (48.4%, OR = 3.76, *p* < 0.001) and positive (66.7%, OR = 8.0, *p* < 0.001) perceptions. This finding was evident for both perception domains. After adjusting for possible confounders, the total perception mean score was a significant predictor of both smartphone use (β = 0.033, *p* < 0.001) and installation of medical applications (β = 0.033, *p* < 0.001). Installation of medical applications was also predicted by physicians (β = 0.771, *p* = 0.024), Table 5.

**Table 4:** Rate of smartphone use (%) and installation of medical applications (%) according to levels of perception towards use

	Use of smartphone				Installation of medical application(s)			
	No.	%	OR [95% CI]	*p*- value	No.	%	OR	*p*- value
Usefulness								
Negative	21	25.6	1		23	28.0	1	
Neutral	93	47.2	2.59[1.47–4.59]	0.001	103	52.0	2.78[1.59–4.85]	< 0.001
Positive	23	74.2	8.35[3.25–21.49]	< 0.001	18	56.3	3.30[1.41–7.71]	0.005
	χ2_LT_ = 23.26, *p* < 0.001				χ2_LT_ = 12.37, *p* < 0.001			
Practicality								
Negative	14	25.9	1		9	16.7	1	
Neutral	99	44.6	2.30[1. 18–4.47]	0.012	112	50.5	5.09[2.38–10.91]	< 0.001
Positive	26	68.4	6.19[2.48–15.47]	< 0.001	25	64.1	8.93[3.39–23.55]	< 0.001
	χ2_LT_ = 16.14, *p* < 0.001				χ2_LT_ = 22.88, *p* < 0.001			
Overall								
Negative	13	26.0	1		10	20.0	1	
Neutral	96	43.2	2.17[1.09–4.30]	0.025	108	48.4	3.76[1.79–7.88]	< 0.001
Positive	26	74.3	8.22[3.07–22.06]	< 0.001	24	66.7	8.00[3.00–21.32]	< 0.001
	χ2_LT_ = 18.44, *p* < 0.001				χ2_LT_ = 19.63, *p* < 0.001			

χ2 --- Chi square for linear trend, *OR*---Odds ratio

**Table 5:** Predictors of use of smartphones and installation of medical applications among HCWs in Saudi Arabia

Independent variables	Use of smartphone			Install med. Applications		
	β	*SE*	*p*-value	β	*SE*	*p*-value
Gender	.278	.39	.474	.600	.390	.124
Age Group	.028	.266	.916	.281	.264	.287
Nationality	.357	.48	.46	−.062	.471	.895
Diploma vs others	−.333	.314	.288	−.354	.311	.255
Single vs others	−.179	.278	.518	.208	.274	.449
physicians vs others	−.432	.331	.192	.771	.342	.024**
Working experience (yrs)	−.040	.029	.172	−.045	.028	.113
Total Perception mean score [%]	.033	.009	<.001**	.033	.008	<.001**
Constant	−1.457	.750	.052	−2.955	.760	.000

**---Statistically significant

## Discussion

Increasing use of smartphone applications among healthcare professionals has gained wide acceptance as a training and information tool [12, 13]. It had been estimated that the percentage of medical professionals using smartphones would reach 66–90% in 2012 [14]. In our study, almost all health providers owned one or more smartphones (96.6%), but only 42.3% utilized it in healthcare practice. A previous survey examining smartphone and its application use was conducted by Accreditation Council for Graduate Medical Education (ACGME) in California [6] showed that more than 85% of respondents used a smartphone, of which the iPhone was the most popular (56%). In the present study, 60.1% reported using other appliances such as; IPAD (43.6%), tablet (7.5%) and others (10.3%).

The most frequently requested application types were textbook/ reference materials (average response = 55%), classification/ treatment algorithms (46%) and general medical knowledge (43%) [6]. In our study, nearly half of all healthcare providers installed applications to use in clinical practice (45.5%). Only 6.1% of all healthcare providers always use the application in their practice, and 26.2% of them use it sometimes. The reasons for use of smartphone in healthcare practice were: as a source of drug information (69.8%), for disease diagnosis (56.4%), to access medical websites (42.5%), to review guideline and protocol related to healthcare (34.1%), for procedure documentation (23.5%), and as a source of patients education materials (22.3%).

### Perception of HCWs towards usefullness and practicality of use of smartphones

Technology Acceptance Model (TAM) was formulated to assess consumer’s level of acceptance of information technology and information system (IT/IS) [2, 7]. In the present study, the results showed that beliefs of healthcare providers in smartphone use - in terms of usefulness and practicality - was less than satisfactory, with only 11% reporting positive attitude. This finding might be attributed to the non-existence of a formal system implemented in the healthcare facilities in Saudi Arabia that allows healthcare providers be convinced of use of smarphone as a new technology that would impact the healthcare quality, by providing practitioners with immediate access to medical and health information.

This technology has resulted in reduced numbers of medical errors, improved decision-making, and upgrading the telemedicine communication among hospital staff [4]. In the present study, use of smartphone in healthcare was reported by less than one-half of all healthcare workers. The use of smartphone in healthcare was predicted by only the level of perception of HCWs towards the practicality and usefulness of its use. Installation of medical applications by HCWs was positively influenced by both the favourable perception as well as the physicians. Nurses’ use of smartphones for work purposes was positively associated with their intention to use smartphones for work purposes, perceived work productivity and perceived quality of care [15]. Prior research has also shown an association between attitude toward HITs and intention to use HITs [16–18]. This finding supports IT consumerization theory and provides empirical support for the argument that nurses’ use of smartphones can improve work productivity [19–21] and enhance the quality of care rendered to patients [19, 22].

This study has some limitations. One of these limitations is that the causal association betweeen the use of smartphone and/or installation of medical applications and other variables could not be guaranteed because of the cross sectional design of the study. Second limitation is that the results could not be generalized being the results of a single health facility.

## Conclusions

In conclusion, smartphone use is not a common practice among HCWs in Saudi Arabia. This could be attiributed to their less than satisfactory level of perception towards the use of smart phones in terms of its usefulness and practicality. There must be a necessary intervention from higher health authorities to enforce the importance of use of smartphones in clinical practice. Conduction of further studies on the impact of smart phone use on the healthcare quality in Saudi Arabia is recommended. Such use can improve HCWs’ perceived work productivity and perceived quality of care rendered to patients.

## Data Availability

Most of the data supporting our findings is contained within the manuscript, and all others, excluding identifying/confidential respondent data should, will be shared upon request. The corresponding author should be contacted to access the data on reasonable request.

## Abbreviations

ACGME: The Accreditation Council for Graduate Medical Education
Apps: Applications
HCWs: Healthcare workers
HIT: Health information technology
IRB: Institutional Review Board
IT: Information technology
IT/IS: Information technology and information system
KAMC: King Abdulaziz Medical city
MNG-HA: Ministry of National Guard-Health Affairs
PEOU: Perceived Ease of Use
PMS: Percentage mean score
PU: Perceived Usefulness
TAM: Technology acceptance model
